# Prospective Exploratory Analysis of Angiogenic Biomarkers in Peripheral Blood in Advanced NSCLC Patients Treated With Bevacizumab Plus Chemotherapy: The ANGIOMET Study

**DOI:** 10.3389/fonc.2021.695038

**Published:** 2021-07-26

**Authors:** Eloisa Jantus-Lewintre, Bartomeu Massutí Sureda, José Luis González Larriba, Delvys Rodríguez-Abreu, Oscar Juan, Ana Blasco, Manuel Dómine, Mariano Provencio Pulla, Javier Garde, Rosa Álvarez, Inmaculada Maestu, Ramón Pérez de Carrión, Ángel Artal, Christian Rolfo, Javier de Castro, Mónica Guillot, Juana Oramas, Ramón de las Peñas, Lioba Ferrera, Natividad Martínez, Òlbia Serra, Rafael Rosell, Carlos Camps

**Affiliations:** ^1^ Departamento de Biotecnología, Universitat Politècnica de València, Unidad Mixta TRIAL, Fundación para la Investigación del Hospital General Universitario de Valencia/Centro de Investigación Príncipe Felipe, CIBERONC, Valencia, Spain; ^2^ Servicio de Oncología Médica, Hospital General Universitario de Alicante, Alicante, Spain; ^3^ Servicio de Oncología Médica, Hospital Clínico Universitario San Carlos, Madrid, Spain; ^4^ Complejo Hospitalario Universitario Insular Materno-Infantil de Gran Canaria, Universidad de las Palmas de Gran Canaria, Las Palmas de Gran Canaria, Spain; ^5^ Servicio de Oncología Médica, Hospital Politécnico y Universitario La Fe, Valencia, Spain; ^6^ Consorcio Hospital General Universitario de Valencia, CIBERONC, Valencia, Spain; ^7^ Servicio de Oncología Médica, Hospital Universitario Fundación Jiménez Díaz, Madrid, Spain; ^8^ Servicio de Oncología Médica, Hospital Universitario Puerta de Hierro, Majadahonda, Spain; ^9^ Departamento de Oncología Médica, Hospital Arnau de Vilanova, Valencia, Spain; ^10^ Departamento de Oncología Médica, Hospital General Universitario Gregorio Marañón, Madrid, Spain; ^11^ Departamento de Oncología Médica, Hospital Universitari Doctor Peset, Valencia, Spain; ^12^ Servicio de Oncología Médica, Hospital Universitario Quirónsalud, Madrid, Spain; ^13^ Servicio de Oncología Médica, Hospital Universitario Miguel Servet, Zaragoza, Spain; ^14^ Experimental Therapeutics Program, Greenbaum Comprehensive Cancer Center, University of Maryland, School of Medicine, Baltimore, MD, United States; ^15^ Servicio de Oncología Médica, Hospital Universitario La Paz, Madrid, Spain; ^16^ Servicio de Oncología Médica, Hospital Son Espases, Palma de Mallorca, Spain; ^17^ Departamento de Oncología Médica, Hospital Universitario de Canarias, Santa Cruz de Tenerife, Spain; ^18^ Departamento de Oncología Médica, Hospital Provincial de Castelló, Castellón, Spain; ^19^ Servicio de Oncología Médica, Hospital Universitario Nuestra Señora de la Candelaria, Santa Cruz de Tenerife, Spain; ^20^ Departamento de Oncología, Hospital General Universitario de Elche, Elche, Spain; ^21^ Departamento de Oncología Médica, Hospital General de l’ Hospitalet, L’Hospitalet de Llobregat, Spain; ^22^ Programa de la Biología del Cáncer y Medicina de Precisión, Institut de Recerca Germans Trias i Pujol, Badalona, Spain/Instituto Oncológico Dr. Rosell, Barcelona, Spain; ^23^ Departamento de Medicina, Universitat Politècnica de València, Unidad Mixta TRIAL, Fundación para la Investigación del Hospital General Universitario de Valencia/Centro de Investigación Príncipe Felipe, CIBERONC, Valencia, Spain

**Keywords:** liquid biopsy, biomarkers, NSCLC, angiogenesis, VEGF

## Abstract

Finding angiogenic prognostic markers in advanced non-small-cell lung cancer is still an unmet medical need. We explored a set of genetic variants in the VEGF-pathway as potential biomarkers to predict clinical outcomes of patients with non-small-cell lung cancer treated with chemotherapy plus bevacizumab. We prospectively analyzed the relationship between VEGF-pathway components with both pathological and prognostic variables in response to chemotherapy plus bevacizumab in 168 patients with non-squamous non-small-cell lung cancer. Circulating levels of VEGF and VEGFR2 and expression of specific endothelial surface markers and single-nucleotide polymorphisms in VEGF-pathway genes were analyzed. The primary clinical endpoint was progression-free survival. Secondary endpoints included overall survival and objective tumor response. *VEGFR-1* rs9582036 variants AA/AC were associated with increased progression-free survival (*p* = 0.012 and *p* = 0.035, respectively), and with improved overall survival (*p* = 0.019) with respect to CC allele. Patients with *VEGF-A* rs3025039 harboring allele TT had also reduced mortality risk (*p* = 0.049) compared with the CC allele. The *VEGF-A* rs833061 variant was found to be related with response to treatment, with 61.1% of patients harboring the CC allele achieving partial treatment response. High pre-treatment circulating levels of VEGF-A were associated with shorter progression-free survival (*p* = 0.036). In conclusion, in this prospective study, genetic variants in *VEGFR-1* and *VEGF-A* and plasma levels of VEGF-A were associated with clinical benefit, progression-free survival, or overall survival in a cohort of advanced non-squamous non-small-cell lung cancer patients receiving chemotherapy plus antiangiogenic therapy.

## Introduction

Lung cancer is one of the most frequent malignancies, and presently the leading cause of cancer-related deaths in Europe (1). Approximately 85% of lung cancer cases are non-small-cell lung cancer (NSCLC), and most NSCLC patients (70%) are diagnosed with advanced-stage disease (i.e., stages IIIB/IV) at presentation (2). A crucial aspect for solid tumor growth is vascularization, and various tumors have been found to produce angiogenic factors themselves or benefit from vascularization induced by inflammatory mediators (3). The major regulator of angiogenesis is vascular endothelial growth factor (VEGF) (4), whose overexpression seems to play a most relevant role in malignant phenotype of solid tumors, including NSCLC (5). Carboplatin-based chemotherapy with bevacizumab has become a standard therapy for eligible NSCLC patients after the results obtained in trials (6–9) and observational studies (10–12). However, careful consideration of the results within the study populations points to the need of determining prognostic markers to select those patients that might achieve greater benefit of treatment with these schemes.

Serum levels of VEGF isoforms and their receptors have been used as prognostic markers and to monitor response to chemotherapy or anti-angiogenic agents (5, 13). In fact, their levels before therapy initiation correlate to prognosis in NSCLC (14–17), but clinical biomarkers and these characteristics alone have been insufficient to predict the course of the disease and response to therapy (16, 17). Variants within *VEGF*-related genes seem to regulate their transcription (18, 19), and several single-nucleotide polymorphisms (SNPs) have been identified, some of them influencing levels of VEGF isoforms or of their receptors in plasma (18, 20–24). Genotype variants in proteins of the VEGF pathway have been shown to impact patient outcomes, however with inconsistent results (25–32), and SNPs are therefore still subject of study as potential prognostic biomarkers. The possible role as biomarkers in NSCLC has also been widely studied in specific endothelial surface proteins CD31, CD34, CD133, and CD146 (33, 34).

Kristen Rat Sarcoma viral oncogene (*KRAS*) is a well-known driver of NSCLC for which no targeted therapy has been developed yet. *KRAS* mutations have been classically defined as a negative prognostic factor in terms of progression-free survival (PFS) and overall survival (OS), but results are heterogenous and the clinical significance remains controversial, also in patients that receive platinum-based chemotherapy (35). However, SNPs in *KRAS* have been related to relapse-free survival in NSCLC (36) and PFS in colorectal cancer (37), and results suggest further research in their potential as biomarkers in NSCLC.

Therefore, the main objective of this clinical study was to increase insight in the correlation between selected molecular biomarkers in genes coding for *VEGF-A*, *VEGF* receptors 1 and 2 (*VEGF-R1* and *VEGFR-2*), and *KRAS*, circulating levels of angiogenic mediators and expression of endothelial markers, and the clinical response to the combined treatment of carboplatin, paclitaxel, and bevacizumab in advanced NSCLC patients.

The authors present the following article in accordance with the STROBE reporting checklist.

## Materials and Methods

ANGIOMET (NCT01814163) was a case-only observational exploratory, post-authorization study. The study has a prospective and multicenter scheme in advanced NSCLC of non-squamous histology, treated in first line with carboplatin-paclitaxel-bevacizumab conducted between February 2011 and February 2013. It was designed to investigate the relationship between genotypes and circulating levels of selected angiogenic mediators and the clinical outcomes and response to this treatment scheme. Twenty hospitals of the public healthcare system across Spain participated.

### Patients and Treatment

The authors are accountable for all aspects of the work in ensuring that questions related to the accuracy or integrity of any part of the work are appropriately investigated and resolved. The trial was conducted in accordance with the Declaration of Helsinki (as revised in 2013). The protocol was approved by the Ethics Committee of Hospital General Universitario de Alicante and was accepted by each participating center. All patients provided written consent for inclusion.

Patients aged over 18 years with advanced non-resectable NSCLC, metastatic or recurrent, and not previously treated with chemotherapy were invited to participate if the treating specialist considered the study therapy as the most appropriate. Those with squamous or non-measurable tumors [according to RECIST 1.1 criteria (38)] or in whom peripheral blood samples could not be obtained were excluded. Detailed inclusion and exclusion criteria are provided in the **Supplementary Appendix**. Recruitment period was 12 months, and the patients were followed for 24 months. These patients received standard therapy of a combination of carboplatin (AUC 6), paclitaxel (200 mg/m^2^), and bevacizumab (15 mg/kg) every 21 days for a total of six cycles unless there was evidence of disease progression or intolerance to treatment.

### Sample Collection and RNA/Protein Measurement

Peripheral blood samples were collected before the first chemotherapy cycle and after three treatment cycles in tubes containing EDTA as anticoagulant (BD Vacutainer^®^, USA) and Blood RNA tubes (PAXgene^®^, USA). Samples were stored at 4°C until DNA, RNA, or plasma extraction was performed.

Blood samples were sent to a reference laboratory within 24 h of blood collection and subject to RNA or plasma extraction. Two centrifugation steps were performed to obtain plasma (10 min at 1,100 g at room temperature and a second centrifugation of the supernatants for 10 min at 2,000 g at RT to eliminate any possible cell fragments). Plasma aliquots were immediately stored at −80°C until further analysis. Circulating levels of VEGF-A and VEGFR-2 in plasma were assayed as previously described (39); double sandwich ELISA (Duo Set, R&D Systems) was used, in which the lower limit of detection for VEGF-A was 31.2 pg/ml, and 15.6 pg/ml for VEGFR-2.

RNA was isolated using the PreAnalytiX blood RNA kit (Qiagen, Valencia, CA, USA) and quantified by spectrophotometry in a NanoDrop2000c device (ThermoScientific, USA). Retrotranscription was performed with 500 ng of RNA using the High-Capacity cDNA Reverse Transcription Kit (Applied Biosystems, USA). Expression of *CD31*, *CD34*, *CD133*, and *CD146* markers was determined by real-time quantitative PCR using primers and probes designed with Taqman^®^ technology (Gene Expression Assays, Applied Biosystems). Expression of target genes was normalized against endogenous expression of a combination of two genes, *GAPDH* and *CDKN1B*, as a reference. cDNA sample from a known cell line was used as a calibrator to minimize inter-trial variability.

To analyze differences between baseline and post-treatment samples, the circulating levels of VEGF-A and VEGFR-2 and the expression of endothelial markers (*CD31*, *CD34*, *CD133*, and *CD146*) were showed as ratios, calculated as the fraction between baseline and post-treatment measurements. Results were dichotomized in “high” and “low.” To evaluate the association with survival times and response to treatment, Chi-square test or Fisher’s Exact test were used as appropriate.

### SNP Selection and Genotyping

Molecular analysis of 10 SNPs in the genes coding for *VEGFR-1*, *VEGFR-2*, *VEGF-A*, and *KRAS* was performed by means of real-time PCR. The SNPs analyzed were *VEGFR-1* rs7996030, *VEGFR-1* rs9582036, *VEGF-A* rs3025039, *VEGF-A* rs833061, *VEGF-A* rs2010963, *VEGFR-2* rs2071559, *VEGFR-2* rs1870377, *KRAS* rs10842513, *KRAS* rs12813551, and *KRAS* rs10505980. Details and rationale for their inclusion in molecular analysis are provided in the supplementary material (**Table S1**).

Genomic DNA was extracted from the buffy coat fraction (EDTA tubes) using QIAamp DNA blood Mini Kit (Qiagen, Valencia, CA, USA). Real-time PCR reactions were carried out on 20 ng of DNA using predesigned assays (TaqMan^®^ SNP Genotyping Assay, Applied Biosystems) and master mix containing DNA polymerase, dNTPs, and buffer (TaqMan^®^ genotyping master mix, Applied Biosystems^®^) in a final volume of 5 µl. A positive and a negative control were included for each SNP analyzed and each reaction plate. All samples were tested in duplicate. The PCR conditions were as follows: 95°C for 1 min, 40 cycles of 95°C for 10 s, 60°C for 1 min. Genotypes were discriminated using probes labeled with FAM and VIC fluorophores in a single multiplex reaction. Fluorescence intensity readings of the probes were measured by the ABI 7900 instrument, and the genotypes were assigned using the SDS 2.4 Software (ABI). All blood samples were centrally analyzed at the Research Institute of Hospital General Universitario de Valencia (Valencia, Spain).

### Endpoints and Variables

Correlation between angiogenic markers and PFS was the primary clinical endpoint (defined as the time from treatment initiation until disease progression or death, whichever occurred first). OS (defined as the time from diagnosis until death from any cause or last clinical follow-up) at 12 and 24 months and response to treatment (classified using the RECIST 1.1. criteria in complete response, partial response, stable disease, and disease progression) were among the secondary endpoints. The absolute and variable frequencies of the responses according to the treatment are presented through contingency tables.

### Statistical Methods

Sample size was estimated considering feasibility in the 20 participating hospitals, given the recruitment period, and set in 200 patients. Descriptive analysis was performed with all variables, which were summarized as mean and standard deviation (SD), median and interquartile range (IQR), or frequency and proportion as appropriate. OS and PFS curves were plotted according to the Kaplan–Meier method, and differences between groups were assessed using the log-rank test. Association of SNPs, circulating levels of angiogenic mediators, and expression of endothelial markers with PFS and OS was analyzed by means of univariate Cox regression models. Multivariate Cox analysis was performed including all statistically significant variables from the univariate analyses. Clinical response to treatment was evaluated by the Chi-square test or Fisher’s Exact test when appropriate. The tests were analyzed using Stata v16.1. software (College Station, TX: StataCorp LLC). The level of significance was set to *a* = 0.05.

## Results

### Patient Characteristics

Of the 201 patients initially included in the study, two of them did not start treatment (consent was withdrawn in one case, and another experienced rapid deterioration); therefore, 199 received the planned combination of carboplatin, paclitaxel, and bevacizumab (CPB). Median age was 62 years (range 54–67), and 139 (70%) were male. Performance status at baseline was ECOG 0 or 1 in the majority (96.2%), and most of them were current smokers or ex-smokers (85.3%). The most frequent histological type was adenocarcinoma (89.1%). Regarding tumor characteristics (**Table 1**), all patients presented a median of two target lesions of mean size 8.41 ± 5.63 cm, and 91.5% presented also non-target lesions. One hundred and seventy-four patients presented two or more metastases, of which pulmonary lymph nodes (65.2%) and lung metastases (50.2%) were the most common (**Table S2**).

**Table 1:** Demographic and clinical characteristics of the study cohort.

N = 201		Mean (SD)	Median (IQR)	n (%)
**Age at baseline**		60.7 (9.16)	62 (54–67)	
**Age range**	<50			29 (14.4%)
	50–59			52 (25.9%)
	60–69			88 (43.8%)
	≥70			32 (15.9%)
**Sex**	Male			62 (30.8%)
	Female			139 (69.2%)
**Performance status**	ECOG 0			44 (24.2%)
	ECOG 1			131 (72.0%)
	ECOG 2			6 (3.3%)
	ECOG 3			1 (0.5%)
**Tobacco smoking history**	Never smoker			29 (14.7%)
	Former smoker			89 (45.2%)
	Packs/year	49.4 (30.6)	40 (30–56)	
	Smoker			79 (40.1%)
	Packs/years	43.6 (21.1)	41 (27–51)	
**Patient previously treated**	Surgery			37 (18.4%)
	Radiotherapy			42 (20.9%)
	Neoadjuvant chemotherapy			5 (2.5%)
	Adjuvant chemotherapy			7 (3.5%)
**No. of target lesions**		2.79 (1.65)	2 (1–9)	201 (100%)
**Size (cm)**		8.41 (5.63)	6.5 (4.6–11.4)	
**No. of non-target lesions**		2.52 (1.28)	2 (1–6)	184 (91.5%)
**Stage**	IIIB			5 (2.5%)
	IV			196 (97.5%)
**Histological type**	Adenocarcinoma			179 (89.1%)
	Large cells			5 (2.5%)
	Poorly differentiated adenocarcinoma			1 (0.5%)
	Others			16 (8.0%)
**TNM classification**		T	N	M
**0**		5 (2.5%)	33 (16.4%)	5 (2.5%)
**1**		21 (10.4%)	10 (5.0%)	196 (97.5%)
**2**		45 (22.4%)	77 (38.3%)	
**3**		32 (15.9%)	57 (28.4%)	
**4**		73 (36.3%)		
**X**		25 (12.4%)	24 (11.9%)	

### Clinical Results

**Figure 1:**
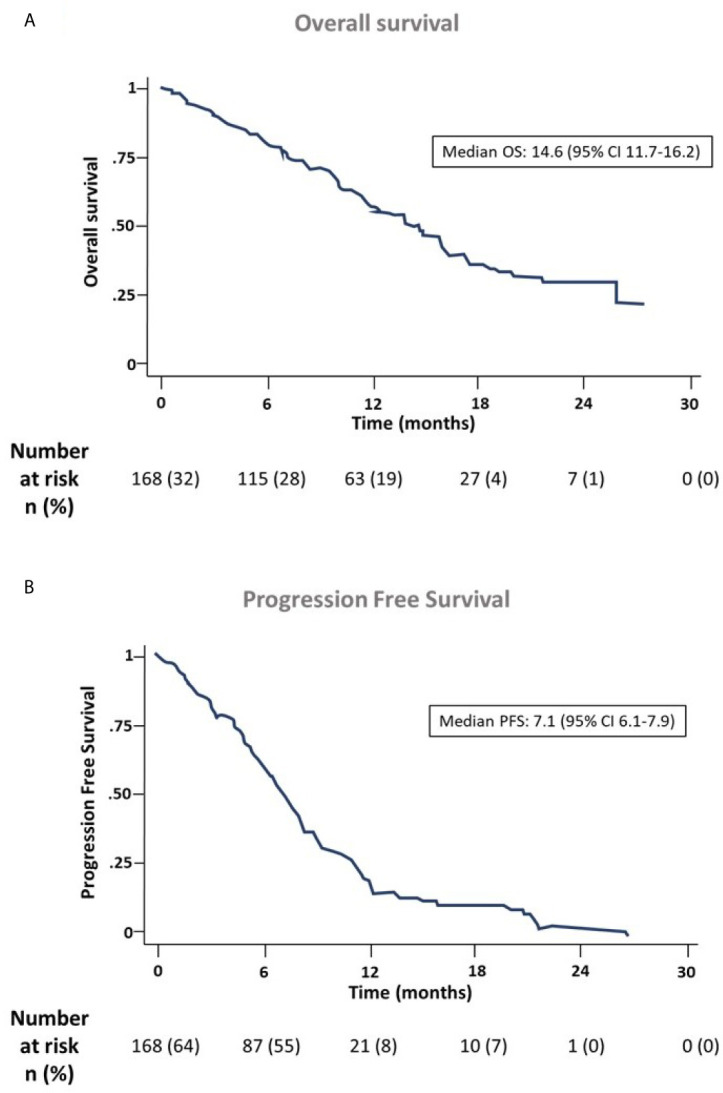
Overall survival **(A)** and progression-free survival **(B)** of the ANGIOMET cohort.

**Figure 2:**
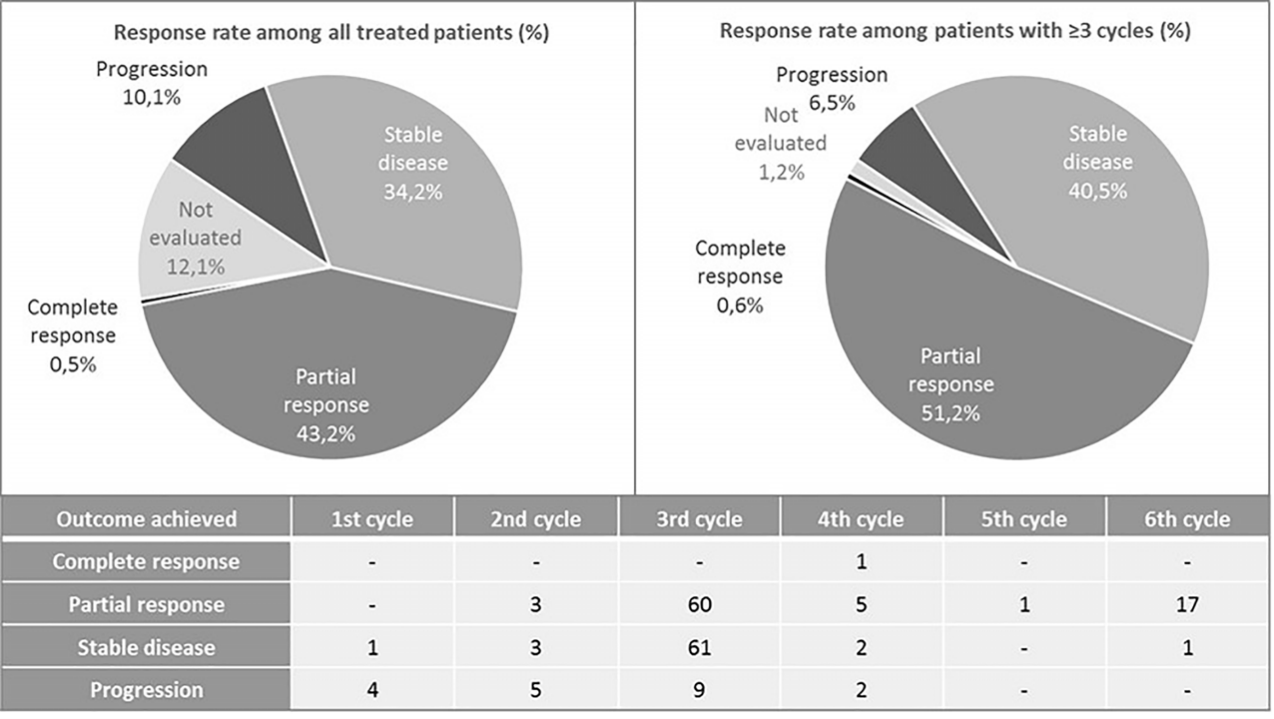
Rate of responses to chemotherapy with carboplatin and paclitaxel combined with bevacizumab.

One hundred and sixty-eight patients had baseline valid blood samples and complete data for the variables of interest and outcomes analyzed. The median OS for these patients was 14.6 months (range 11.7–16.2) (**Figure 1A**), and the median PFS was 7.1 months (range 6.1–7.9) (**Figure 1B**). The OS was 80% at 6 months and 58% at 1 year. Regarding PFS, it was 59% at 6 months, and 18% after 1 year. One patient achieved complete response, 86 achieved partial response (43.2% of the overall sample and 51.2% among those that received at least three cycles), and 20 (10.1%) progressed during initial therapy (**Figure 2**). All patients were followed up until death, abandonment, or end of study. Of the 199 treated patients, 11 (5.5%) died during initial therapy and 142 (71.3%) during the follow-up period. Adverse events and other safety outcomes are shown in **Supplementary Appendix** and **Tables S3–S5**.

### Genetic Variants and Outcomes

Molecular analysis of genetic variants was performed in DNA samples obtained from peripheral blood, and the frequencies obtained are detailed in **Table 2**. **Table 3** shows the results on the association between SNPs in *VEGFA*, *VEGFR1*, *VEGFR2*, and *KRAS* genes and treatment response. Among the multiple variants analyzed, only *VEGF-A* rs833061 was significantly related with a clinical response to CPB (*p *= 0.036). Patients with the CT genotype had a higher percentage of partial response to treatment compared with patients with CC and TT alleles (61.1 *vs.* 38.9 and 40.5% respectively).

**Table 2:** Genotype data and SNPs frequency.

Gene	RF number	Alleles	Frequency n (%) (N = 168)
*VEGFR-1*	rs9582036	CC	17 (10.1%)
		AA	74 (44.1%)
		AC	77 (45.8%)
	rs7996030	GG	95 (56.6%)
		AA	7 (4.2%)
		AG	66 (39.3%)
*VEGFR-2*	rs2071559	AA	46 (27.4%)
		GG	41 (24.4%)
		AG	81 (48.2%)
	rs870377	AA	8 (4.8%)
		TT	101 (60.1%)
		AT	59 (35.1%)
*VEGF-A*	rs3025039	CC	121 (72%)
		TT	21 (12.5%)
		CT	26 (15.5%)
	rs833061	CC	44 (26.2%)
		TT	41 (24.4%)
		CT	83 (49.4%)
	rs2010963	CC	11 (6.6%)
		GG	79 (47%)
		CG	78 (46.4%)
*KRAS*	rs10842513	CC	143 (85.1%)
		TT	2 (1.2%)
		CT	23 (13.7%)
	rs12813551	CC	16 (9.5%)
		TT	66 (39.3%)
		CT	86 (51.2%)
	rs10505980	CC	73 (43.5%)
		TT	18 (10.7%)
		CT	77 (45.8%)

**Table 3:** Association of SNPs in *VEGFA*, *VEGFR1*, *VEGFR2*, and *KRAS* genes with response to BCP therapy.

Gene	SNP	Alleles	RESPONSE TO TREATMENT				
			Disease progression	Stable disease	Partial response	Complete response	p-value
*VEGFR-1*	rs9582036	CC	0 (0.0%)	6 (46.2%)	7 (53.9%)	0 (0.0%)	0.159
		AA	9 (14.3%)	29 (46.0%)	25 (39.7%)	0 (0.0%)	
		AC	6 (8.7%)	21 (30.4%)	41 (59.4%)	1 (1.5%)	
	rs7996030	GG	9 (11.3%)	32 (40.0%)	38 (47.5%)	1 (1.3%)	0.909
		AA	0 (0.0%)	1 (20.0%)	4 (80.0%)	0 (0.0%)	
		AG	6 (10.0%)	23 (38.3%)	31 (51.7%)	0 (0.0%)	
*VEGFR-2*	rs2071559	AA	0 (0.0%)	1 (20.0%)	4 (80.0%)	0 (0.0%)	0.909
		GG	9 (11.3%)	32 (40.0%)	38 (47.5%)	1 (1.3%)	
		AG	6 (10.0%)	23 (38.3%)	31 (51.7%)	0 (0.0%)	
	rs870377	AA	1 (16.7%)	3 (50.0%)	2 (33.3%)	0 (0.0%)	0.852
		TT	8 (8.9%)	34 (37.8%)	47 (52.2%)	1 (1.1%)	
		AT	6 (12.2%)	19 (38.8%)	24 (49.0%)	0 (0.0%)	
*VEGF-A*	rs3025039	CC	12 (11.8%)	37 (36.3%)	52 (51.0%)	1 (1.0%)	0.937
		TT	1 (5.3%)	9 (47.4%)	9 (47.4%)	0 (0.0%)	
		CT	2 (8.3%)	10 (41.7%)	12 (50.0%)	0 (0.0%)	
	rs833061	CC	3 (8.3%)	19 (52.8%)	14 (38.9%)	0 (0.0%)	**0.036**
		TT	3 (8.1%)	19 (51.4%)	15 (40.5%)	0 (0.0%)	
		CT	9 (12.5%)	18 (25.0%)	44 (61.1%)	1 (1.4%)	
	rs2010963	CC	0 (0.0%)	5 (50.0%)	5 (50.0%)	0 (0.0%)	0.565
		GG	8 (12.5%)	27 (42.2%)	28 (43.8%)	1 (1.6%)	
		CG	7 (9.9%)	24 (33.8%)	40 (56.3%)	0 (0.0%)	
*KRAS*	rs10842513	CC	13 (10.5%)	45 (36.3%)	65 (52.4%)	1 (0.8%)	0.328
		TT	0 (0.0%)	0 (0.0%)	2 (100.0%)	0 (0.0%)	
		CT	2 (10.5%)	11 (57.9%)	6 (31.6%)	0 (0.0%)	
	rs12813551	CC	1 (6.3%)	4 (25.0%)	11 (68.8%)	0 (0.0%)	0.168
		TT	4 (7.0%)	29 (50.9%)	24 (42.1%)	0 (0.0%)	
		CT	10 (13.9%)	23 (31.9%)	38 (52.8%)	1 (1.4%)	
	rs10505980	CC	5 (7.9%)	31 (49.2%)	27 (42.9%)	0 (0.0%)	0.216
		TT	1 (6.3%)	4 (25.0%)	11 (68.8%)	0 (0.0%)	
		CT	9 (13.6%)	21 (31.8%)	35 (53.0%)	1 (1.5%)	

P-values marked with bold indicate statistically significant p-values.

**Figure 3:**
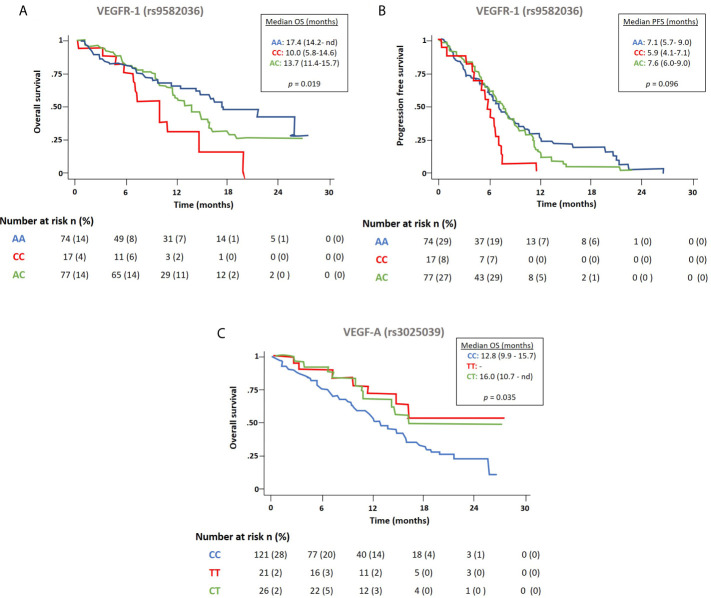
Effect of the polymorphism in *VEGFR-1* rs9582036 in estimated OS **(A)** and PFS **(B)**, and in *VEGFA* rs3025039 in estimated OS **(C)**.

Correlation between analyzed polymorphisms and PFS or OS in our cohort is shown in **Table 4**. The genetic variant in *VEGFR-1* rs9582036 was significantly associated to OS and PFS and *VEGF-A* rs3025039 was significantly associated to OS (**Figure 3**). Patients harboring the AA/AC alleles in *VEGFR-1* rs9582036 had increased OS compared with those with CC allele, with median OS of 17.4, 13.7 and 10.0 months, respectively. Similarly, patients with the *VEGF-A* rs3025039 SNPs, TT and CT presented half the risk of death than those patients with the CC allele (HR_TT_: 0.45, *p* = 0.049; HR_CT:_ 0.54, *p* = 0.068). On the other hand, the PFS was significatively increased in patients with *VEGFR-1* rs9582036 AA/AC alleles (HR_AA_: 0.47, *p* = 0.012; HR_AC_: 0.54, *p* = 0.035). No association was found in any of the SNPs studied in *KRAS* and OS or PFS (**Table 4**).

**Table 4:** Association of SNPs in *VEGFA*, *VEGFR1*, *VEGFR2*, and *KRAS* genes with OS and PFS.

Gene	RF number	Alleles	OS		PFS	
			HR (CI 95%)	*p*	HR (CI 95%)	*p*
*VEGFR-1*	rs9582036	CC	REF	–	–	–
		AA	0.39 (0.20 - 0.78)	**0.007**	0.47 (0.26 - 0.85)	**0.012**
		AC	0.54 (0.29 – 1.03)	0.060	0.54 (0.30 – 0.96)	**0.035**
	rs7996030	GG	REF	–	–	–
		AA	1.29 (0.40 - 4.18)	0.674	1.82 (0.72 - 4.58)	0.206
		AG	1.25 (0.80 - 1.94)	0.328	1.31 (0.92 – 1.87)	0.134
*VEGFR-2*	rs2071559	AA	REF	–	–	–
		GG	1.42 (0.77 - 2.63)	0.260	1.40 (0.86 - 2.27)	0.175
		AG	1.23 (0.74 – 2.05)	0.423	1.16 (0.77 - 1.74)	0.473
	rs870377	AA	REF	–	–	–
		TT	0.70 (0.30 - 1.65)	0.418	0.77 (0.35 - 1.68)	0.512
		AT	0.85 (0.35 - 2.03)	0.713	0.83 (0.38 - 1.84)	0.648
*VEGF-A*	rs3025039	CC	REF	–	–	–
		TT	0.45 (0.21 - 0.99)	**0.048**	(0.59 - 1.61)	0.917
		CT	0.54 (0.28 – 1.05)	0.068	(0.43 – 1.16)	0.172
	rs833061	CC	REF	–	–	–
		TT	0.88 (0.50 - 1.56)	0.666	0.86 (0.53 - 1.40)	0.82
		CT	0.65 (0.38 - 1.08)	0.097	0.82 (0.54 – 1.24)	0.354
	rs2010963	CC	REF	–	–	**-**
		GG	1.59 (0.57 - 4.45)	0.378	0.89 (0.42 - 1.87)	0.762
		CG	1.52 (0.55 – 4.26)	0.421	0.77 (0.36 – 1.62)	0.484
*KRAS*	rs10842513	CC	REF	–	–	–
		TT	0.81 (0.11 - 5.87)	0.836	1.15 (0.28 - 4.69)	0.841
		CT	1.26 (0.69 – 2.28)	0.455	1.39 (0.85 - 2.27)	0.192
	rs12813551	CC	REF	–	–	–
		TT	0.44 (0.17 - 1.14)	0.092	0.86 (0.46 - 1.60)	0.635
		CT	0.91 (0.58 – 1.41)	0.662	0.95 (0.66 - 1.37)	0.773
	rs10505980	CC	REF	–	–	–
		TT	0.65 (0.29 - 1.47)	0.288	1.32 (0.76 - 2.31)	0.328
		CT	0.86 (0.55 – 1.35)	0.517	0.93 (0.64 - 1.34)	0.693

Multiple Cox-regression allelic model for PFS and OS was performed.

P-values marked with bold indicate statistically significant p-values.

### Circulating Levels of VEGF-A and VEGFR-2 and Clinical Outcomes

We evaluated baseline VEGF-A and VEGFR-2 circulating levels as possible biomarkers of clinical outcomes such as PFS, OS, or treatment response to CPB (**Table S6**). By using the median of each soluble marker as a cutoff, continuous variables were dichotomized as “high” (values above the median) and “low” (values equal or less than the median). Our results showed that patients within the “high” VEGF-A group had significantly shorter PFS [5.9 *vs.* 8.4 months, *p* = 0.036] than patients within the “low” VEGF-A group. A similar trend (non-statistically significant) was observed in OS (12.0 *vs.* 16.0 months, *p* = 0.095). Survival analysis was performed also for VEGFR-2, and in this case, no significant association was found between basal levels of this biomarker and PFS or OS.

When we analyzed the possible changes in circulating levels of VEGF-A and VEGFR-2 between pre- and post-treatment values (expressed as a ratio and using a cutoff value = 1), we found no significant association with risk of death, progression, or response to treatment either in the group with a ratio below 1 or in the group with a ratio above 1 (data not shown).

### Expression of Endothelial Markers and Prognosis

No correlations were found between expression levels of *CD31*, *CD34*, *CD146*, or *CD133* at baseline, post treatment, or when ratios baseline/post-treatment (cutoff value = median) were analyzed and correlated with PFS, OS, or response to treatment in this study (**Table S6** and data not shown).

### Multivariate Analysis

A multivariate Cox regression model for PFS and OS was built using clinical variables (PS-ECOG, gender, histology, smoking habit) and the analytical variables that were found significant in the univariate analysis. Regarding PFS, this analysis revealed that only the *VEGFR-1* rs9582036 and baseline circulating levels of VEGF-A were independent prognostic variables (*p* = 0.012 and p = 0.042, respectively) in our patient population. Moreover, ECOG-PS, *VEGFR-1* rs9582036, and *VEGF-A* rs3025039 were found as independent markers for OS (*p* = 0.004, *p* = 0.018 and *p* = 0.007, respectively) in our study.

## Discussion

There is a strong unmet need for new biomarkers that can predict clinical responses to various therapies in patients with NSCLC, facilitating a more individualized treatment. During the last years, several studies have evaluated different molecules related to VEGF pathway in relation to clinical outcomes in patients with NSCLC treated with CPB regimen, but until now, there are no validated predictive biomarkers in the clinical setting (40). This prospective study contributes to the body of evidence demonstrating the potential of some biomarkers, including SNPs in angiogenic genes and circulating levels of VEGF, as prognostic factors in NSCLC. Our results show that both *VEGFR-1* rs9582036 and *VEGF-A* rs3025039 were independent prognostic markers for OS. Moreover, shorter PFS correlates with higher baseline plasma levels of VEGF-A and the presence of the CC allele in *VEGFR-1* rs9582036. Then, we propose the analysis of variants in *VEGFR-1* and *VEGF-A* as potential biomarkers in patients with NSCLC treated with CPB.

Several studies have linked rs9582036 *VEGFR-1* polymorphism and clinical outcomes in bevacizumab-treated patients with different kinds of cancer (36, 41, 42). The CC allele of rs9582036 variant was predictive of shorter survival in pancreatic and colorectal cancer (41, 42). Regarding NSCLC patients, the study performed by Glubb et al. (36) in 2015 showed that AA variants of rs9582036 were associated with longer relapse-free survival independent of the treatment, and were proposed as a prognostic biomarker in stage I-III NSCLC (36). Our results found that the CC rs9582036 allele has a negative predictive effect in patients with advanced NSCLC treated with chemotherapy plus bevacizumab, while the AA and AC variants are associated with an increase in PFS and OS. Taken together, these data suggest that carriers of the CC allele of the *VEGFR-1* variant rs9582036 may be less responsive to angiogenesis inhibition having a detrimental effect on survival. However, one should consider that the *VEGFR-1* variants found in our study were detected in peripheral blood, while the SNPs reported in the study by Glubb et al. (36) were found in somatic tumor cells. Considering all these pieces of evidence supporting the role of the rs9582036 polymorphism in *VEGFR-1* in treatment response and outcome in NSCLC, more studies are needed to understand the molecular mechanism of *VEGFR-1* genetic variation in response to VEGF blockage.

Additionally, we found that patients harboring both the TT and CT alleles in rs3025039 *VEGF-A* had reduced their risk of death by approximately half compared to CC. Again, there is some controversy in the literature regarding the effect of this SNP in lung cancer clinical outcomes. While patients with advance NSCLC treated with chemoradiotherapy showed no significant association with survival (43), a trend toward improved survival was observed in early-stage NSCLC patients treated with surgical resection, in concordance with our findings (30). In line with these results, Chen et al. reported that patients with the TT allele treated with chemotherapy presented lower risk of death from all causes (44).

Only *VEGF-A* rs833061 polymorphism was found to be significantly associated with response to treatment in our study. Previous reports have already demonstrated a potential role of this particular SNP in the metastatic capacity of many tumors, including NSCLC (26, 30, 45–47). Regarding its impact on the therapeutic efficiency of bevacizumab plus chemotherapy, our analysis showed that more than 60% of the rs833061 CT carriers had a clinical benefit of the treatment. That agrees with the exploratory study performed by Pallaud et al. in 2014 (27), showing increased best overall response to first-line bevacizumab plus chemotherapy treatment in patients harboring this allele. These data provide strong evidence of the predictive value of rs833061 SNP as potential biomarker for response to anti-VEGF therapy.

We also investigated whether expression profiles of circulating VEGF-A and VEGFR-2 could be of clinical interest for finding predictors of clinical outcomes to CPB in NSCLC. We found that high pre-treatment VEGF-A plasma levels were associated with less favorable prognosis, increasing the risk of progression. In recent years, some studies have also explored the usefulness of measuring the levels of angiogenic factors as prognostic or predictive markers in NSCLC (25–28, 30, 31, 36, 48). Sanmartín et al. identified a signature of angiogenic factors related with NSCLC outcome (48). Particularly, patients with high levels of VEGF-A and low expression of VEGF-B and VEGF-D had worse OS and lower relapse-free survival. A metanalysis including 74 studies and 7,631 patients found that *VEGF-A* overexpression was an independent prognostic factor in early-stage NSCLC (49). Regarding the role of circulating levels of VEGF-A, there are evidences showing an association between higher levels of this angiogenic mediator and worse clinical outcomes in NSCLC (49, 50). All together, these results suggest that high levels of *VEGF-A* could be associated with negative outcomes in NSCLC. Since *VEGF-A* is a target of bevacizumab treatment, one possible explanation of the effects of higher concentrations of *VEGF-A* in peripheral blood is directly related to a lower therapeutic effect of this drug. Evaluation of *VEGF* levels in lung cancer development and in response to therapy might suppose an important tool to better understand its prognostic impact.

The limitations of this study include its exploratory nature, since the sample size followed the feasibility criteria and is limited. Also, the SNP panel used is limited, and it should be extended in order to test other SNPs, specifically in *VEGF-A*, that have shown an association with clinical outcomes in other cancers. On the other hand, this is one of the few studies that prospectively evaluated the effect of different angiogenesis biomarkers in liquid biopsies in the NSCLC setting, accurately collecting information and eliminating any recall bias.

The results of this prospective study support previous findings regarding the influence of SNPs on angiogenic VEGF genes, and the circulating levels of VEGF on the clinical outcomes of patients with non-squamous NSCLC receiving antiangiogenic treatment such as bevacizumab. In most cases, consistent results across studies are lacking, warranting further research on the subject before molecular biomarkers can be useful in selecting patients with NSCLC and being included in treatment algorithms for antiangiogenic agents. However, important advances are being made in other cancer types, including breast cancer, in which a recent study has found an association of independent prognostic factors with specific treatments and has weighted them by the outcome category (51).

## Data Availability Statement

The raw data supporting the conclusions of this article will be made available by the authors, without undue reservation.

## Ethics Statement

The studies involving human participants were reviewed and approved by Hospital General Universitario de Alicante. The patients/participants provided their written informed consent to participate in this study.

## Author Contributions

Conception and design: CC, BM, JC, and EJ-L. Administrative support: CC, BM, and RR. Provision of study materials or patients: JGL, DR-A, OJ, AB, MD, MP, JG, RÁ, IM, RPC, AA, CR, JC, MG, JO, RP, LF, NM, and ÒS. Collection and assembly of data: EJ-L, JGL, DR-A, OJ, AB, MD, MP, JG, RÁ, IM, RPC, AA, CR, JC, MG, JO, RP, LF, NM, and ÒS. Data analysis and interpretation: EJ-L, CC, BM, and RR. Manuscript writing: all authors. All authors contributed to the article and approved the submitted version.

## Conflict of Interest

DR-A declares having received honoraria for lectures and advisory boards from Bristol-Myers-Squibb, Merck Sharp & Dohme, Hoffmann-La Roche, Pierre-Fabre, Novartis, Boehringer, Pfizer, Lilly, and AstraZeneca. OJ declares that he has received payment for advisory services from Boehringer Ingelheim, Bristol-Myers Squibb, Merck Sharp & Dohme, Roche/Genetech, AstraZeneca, Pfizer, Eli Lilly, Abbvie, and Takeda. MP reports having received payments from Bristol-Myers Squibb, AstraZeneca, Takeda, Merck Sharp & Dohme, Hoffmann-La Roche, and Novartis for advisory services. JC received fees for advisory services from Hoffman-La Roche. CR declares having received lecture fees from Merck Sharp & Dohme and AstraZeneca, advisory board payments from Archer, Iceivafa, Merck Sharp & Dohme and Laboratorios Serono, and honoraria for consultancy roles from Mylan and Oncompass.

The remaining authors declare that the research was conducted in the absence of any commercial or financial relationships that could be construed as a potential conflict of interest.

## Publisher’s Note

All claims expressed in this article are solely those of the authors and do not necessarily represent those of their affiliated organizations, or those of the publisher, the editors and the reviewers. Any product that may be evaluated in this article, or claim that may be made by its manufacturer, is not guaranteed or endorsed by the publisher.

## Abbreviations

CPB, Carboplatin, paclitaxel, and bevacizumab; IQR, Interquartile range; KRAS, Kristen Rat Sarcoma viral oncogene; NSCLC, Non-small-cell lung cancer; OS, Overall survival; PFS, Progression-free survival; SD, Standard deviation; SNP, Single-nucleotide polymorphism.
