# Supplementary material for: Prospective Exploratory Analysis of Angiogenic Biomarkers in Peripheral Blood in Advanced NSCLC Patients Treated With Bevacizumab Plus Chemotherapy: The ANGIOMET Study
Source: Front Oncol. 2021 Jul 26;11:695038. doi: 10.3389/fonc.2021.695038 (PMC8350788; doi:10.3389/fonc.2021.695038)
Supplement: Supplementary file 1 [file DataSheet_1.docx]

Supplementary Material

Table of Contents

[Materials and Methods 2](#_Toc55226511)

[1. Detailed inclusion and exclusion criteria 2](#_Toc55226512)

[Inclusion criteria 2](#_Toc55226513)

[Exclusion criteria 2](#_Toc55226514)

[2. Treatment characteristics 3](#_Toc55226515)

[3. Safety analysis 3](#_Toc55226516)

[Tables 4](#_Toc55226517)

[Table S1. Single-nucleotide polymorphisms selected 4](#_Toc55226518)

[Table S2. Number and locations of metastases 7](#_Toc55226519)

[Table S4. Cycles of initial therapy received and causes of interruption 9](#_Toc55226520)

[Table S5. Adverse events classified according to CTC-AE 4.0 10](#_Toc55226521)

[Table S6. Association between VEGF, VEGFR-2, CD31, CD34, CD133, CD146 levels and objective response to treatment 12](#_Toc55226522)

# Materials and Methods

## 1. Detailed inclusion and exclusion criteria

### Inclusion criteria

- Written informed consent to participate in the study, obtained before patient inclusion, in which extraction of samples of peripheral blood for subsequent molecular analysis is specifically authorized.
- Age older than 18 years.
- Diagnosis of advanced non-resectable, metastatic or recurrent non-small cell lung cancer with a histological type of non-squamous cell, histologically or cytologically documented.
- Patient is candidate to receive a first line of treatment and the treating specialist/researcher considers that the most appropriate option is the combination of carboplatin, paclitaxel and bevacizumab, according to the respective specifications of these drugs. The decision is always independent of the patient’s participation in the study.
- Measurable tumor, according to the RECIST criteria.
- Possibility of taking peripheral blood samples (at least 30 ml).
- Patient is able to adhere to the study requirements.
- Blood test results performed within 2 weeks prior to inclusion:
- Absolute neutrophil count ANC > 1500 / μl.
- Platelets > 100,000 / μl.
- Hemoglobin > 10 g / l.
- Total bilirubin < 1.5 mg / dl.
- Serum GOT / GPT = 3 × ULN, except in case of known liver metastases (>5 × LSN).
- Creatinine clearance ≥ 45 ml / min.

### Exclusion criteria

- Previous chemotherapy for advanced disease (allowed if used in adjuvant and/or neoadjuvant context according to initial diagnosis)
- Patients with a history of hemoptysis (½ teaspoon of red blood within three months prior to inclusion)
- Tumor invasion of large vessels evidenced by means of imaging techniques.
- Untreated brain metastases.
- Major surgical procedures, open biopsies or major traumatic injury within 28 days prior to the treatment initiation or a major surgical procedure during the course of the study is anticipated.
- Minor surgical procedures within 24 hours before the start of treatment.
- Uncontrolled hypertension (systolic blood pressure > 150 mm Hg and / or diastolic blood pressure > 100 mm Hg)
- Clinically active cardiovascular disease, such as: cerebrovascular accident (within 6 months prior to inclusion), myocardial infarction (within 6 months prior to inclusion), unstable angina (according to NYHA grade II or greater), congestive heart failure, severe arrhythmia requiring a pharmacological treatment during the study or that might interfere with chemotherapy.
- Ulcerations or fractures considered important.

## 2. Treatment characteristics

The median number of treatment cycles delivered was 5: overall, 31 patients (15.6%) received less than 3 cycles and 97 (48.7%) received the full planned combined therapy (6 cycles) and continued with maintenance with bevacizumab. **Ninety-seven (48.7%) patients received the full 6-cycle initial therapy, while 102 (51.3%) interrupted at a given cycle. The majority of those that abandoned received ≥3 cycles (*n* = 71, 69.6%). The most frequent reasons for interruption were investigator decision (15.6%), toxicity (15.6%) or disease progression (11.1%).**

## 3. Safety analysis

A total of 825 AEs were experienced by 193 (97.1%) patients reported adverse events, and AEs in 183 patients were considered related to study medication. Severe events (n= 155) occurred in 88 patients (44.0%), and 89 events (57.1%) were considered as related to the study medication. The most frequent AEs were gastrointestinal disorders (60.8%; 12 patients of 121 grade 3), blood disorders (52.8%, mainly anemia 51.3%; 17 of 105 grade 3, and 2 grade 4; 6 cases of grade III-IV febrile neutropenia were reported), general disorders and administration site conditions (56.8%; 13 of 113 grade 3 and 1 grade 4, mostly fatigue), investigations (53.3%; 25 of 106 grade 3 and 12 grade 4, consisting of neutropenia and thrombopenia), nervous system disorders (45.7%; 15 of 91 grade 3 and 1 grade 4), skin disorders (44.2%; 2 of 88 grade 3) and respiratory disorders (33.2%; all mild or moderate). Vascular disorders occurred in 27 (13.6) patients, of which 18 (9.0) reported mild to moderate hypertension and 3 (1.5%) hypertensive crises. Allergic reactions and infections were infrequent (3.0% and 5.5%, respectively) but 3 severe allergic reactions and 2 severe infections were recorded. One acute heart failure grade IV was recorded. Additional details can be found in **Table S5**.

# Tables

## Table S1. Single-nucleotide polymorphisms selected

|  |  |  |  | | | | | |
| --- | --- | --- | --- | --- | --- | --- | --- | --- |
| Gene | Protein | rs number | | **Variant** | **Assay ID** | **MAF** | **HWE** | **Rationale for genotyping in NCLC patients** |
| *FLT-1* | VEGFR-1 | rs7996030 | | G>A | C__11505993_10 | 0.24 | 0.08/0.20 | Associated to relapse-free survival in NSCLC(1) |
| *FLT-1* | VEGFR-1 | rs9582036 | | A<C | C___1910658_10 | 0.29 | 0.15-0.02 | Associated to relapse-free survival (1, 2)and overall survival in NSCLC (2) |
| *VEGFA* | VEGF-A | rs3025039 | | C>T | C__16198794_10 | 0.17 | 0.54 | Putative function |
| *VEGFA* | VEGF-A | rs833061 | | T>C | C___1647381_10 | 0.47 |  | Association with lower levels of VEGF(3), included in Heist et al.(4) |
| *VEGFA* | VEGF-A | rs2010963 | | G>C | C___8311614_10 | 0.27 | 0.13 | Association with survival in NSCLC(4) |
| *KDR* | VEGFR-2 | rs2071559 | | C>T | C__15869271_10 | 0.50 | 0.32 | Evidence of function (5) |
| *KDR* | VEGFR-2 | rs1870377 | | T>A | C__11895315_20 | 0.28 | 0.30 | Evidence of function (5) |
| *KRAS* | KRAS | rs10842513 | | C>T | C__31385754_10 | 0.11 | 0.002/0.11 | - Lymphoblastoid cell lines and population specificity of cis expression quantitative trait loci (6)  - Associated to relapse-free survival in NSCLC (1) |
| *KRAS* | KRAS | rs12813551 | | T>C | C__31385887_10 | 0.41 | 0.29/0.60 | - Lymphoblastoid cell lines and population specificity of cis expression quantitative trait loci(6)  - Associated to relapse-free survival in NSCLC (1) |
| *KRAS* | KRAS | rs10505980 | | C>T | C__29938457_20 | 0.39 | 0.38 | -Associated to relapse-free survival in NSCL (1) |
|  |  |  | *MAF, minor allele frequency at European MAF (CEU HapMap population); HWE, Hardy Weinberg equilibrium in the HapMap CEU population.*  *^a^Included in Glubb et al.(1), putative function predicted by FuncPred*  1. Glubb DM, Pare-Brunet L, Jantus-Lewintre E, Jiang C, Crona D, Etheridge AS, et al. Functional FLT1 Genetic Variation is a Prognostic Factor for Recurrence in Stage I-III Non-Small-Cell Lung Cancer. Journal of thoracic oncology : official publication of the International Association for the Study of Lung Cancer. 2015;10:1067-75.  2. Lambrechts D, Claes B, Delmar P, Reumers J, Mazzone M, Yesilyurt BT, et al. VEGF pathway genetic variants as biomarkers of treatment outcome with bevacizumab: an analysis of data from the AViTA and AVOREN randomised trials. The Lancet Oncology. 2012;13:724-33.  3. Stevens A, Soden J, Brenchley PE, Ralph S, Ray DW. Haplotype analysis of the polymorphic human vascular endothelial growth factor gene promoter. Cancer research. 2003;63:812-6.  4. Heist RS, Zhai R, Liu G, Zhou W, Lin X, Su L, et al. VEGF polymorphisms and survival in early-stage non-small-cell lung cancer. Journal of clinical oncology : official journal of the American Society of Clinical Oncology. 2008;26:856-62.  5. Glubb DM, Cerri E, Giese A, Zhang W, Mirza O, Thompson EE, et al. Novel functional germline variants in the VEGF receptor 2 gene and their effect on gene expression and microvessel density in lung cancer. Clinical cancer research : an official journal of the American Association for Cancer Research. 2011;17:5257-67.  6. Pare-Brunet L, Glubb D, Evans P, Berenguer-Llergo A, Etheridge AS, Skol AD, et al. Discovery and functional assessment of gene variants in the vascular endothelial growth factor pathway. Human mutation. 2014;35:227-35. | | | | | |

## Table S2. Number and locations of metastases

|  | No. of patients | No. of metastases | | | |
| --- | --- | --- | --- | --- | --- |
|  |  | **1** | **2** | **3** | **≥4** |
| Metastasis | 200 (99.5%) | 26 | 65 | 63 | 46 |
| Pulmonary lymph nodes | 131 (65.2%) | 6 | 43 | 46 | 36 |
| Lung | 101 (50.2%) | 9 | 26 | 35 | 31 |
| Bone | 76 (37.8%) | 6 | 12 | 27 | 31 |
| Pleural effusion | 46 (22.9%) | 2 | 11 | 13 | 20 |
| Liver | 33 (16.4%) | - | 6 | 16 | 11 |
| Kidney | 33 (16.4%) | - | 5 | 12 | 16 |
| Central Nervous System | 25 (12.4%) | 2 | 5 | 10 | 8 |
| Pleura | 23 (11.4%) | 1 | 9 | 9 | 4 |
| Extrapulmonary lymph nodes | 29 (14.5%) | - | 5 | 12 | 12 |
| Pericardial effusion | 11 (5.5%) | - | - | - | 11 |
| Skin / Soft Tissues | 10 (5.0%) | - | 2 | 2 | 6 |
| Muscle | 1 (0.5%) | - | - | - | 1 |
| Other locations | 23 (11.4%) | - | 6 | 7 | 10 |

**Table S3. Adverse events**

| Total AE  (n= 198, 1 missing) | Patients experiencing AE | Grade* | | | |
| --- | --- | --- | --- | --- | --- |
|  |  | **1** | **2** | **3** | **4** |
| Any AE | 193 (97.1%) | 26 (13.5%) | 77 (39.9%) | 70 (36.3%) | 20 (10.4%) |
| Related to therapy | 42 (21.1%) | 27 (14.8%) | 75 (41%) | 63 (34.4%) | 18 (9.8%) |
| Unrelated to therapy | 10 (5.0%) | 72 (47.7%) | 57 (37.7%) | 20 (13.2%) | 2 (1.3%) |
| Both | 141 (70.9%) |  | | | |
| *AE listed as “both” (related or unrelated to therapy) have been distributed in the corresponding lines to describe severity* | | | | | |

## Table S4. Cycles of initial therapy received and causes of interruption

|  | | | No. of patients that interrupt at: | | | | |
| --- | --- | --- | --- | --- | --- | --- | --- |
| N = 199 | | **Total** | **Cycle 1** | **Cycle 2** | **Cycle 3** | **Cycle 4** | **Cycle 5** |
| Initial therapy complete | | **97 (48,7%)** |  |  |  |  |  |
| Initial therapy interrupted | | **102 (51,3%)** | 23 (11.6%) | 8 (4.5%) | 17 (10.1%) | 37 (24.5%) | 17 (14.9%) |
| Cumulative interruption | |  | 11.6% | 15.6% | 24.1% | 42.7% | 51.3% |
| Reasons | **Investigator decision** | 31 (15,6%) | 7 | 0 | 3 | 16 | 5 |
|  | **Patient decision** | 3 (1,5%) | 1 | 0 | 0 | 2 | 0 |
|  | **Promoter decision** | 1 (0,5%) | 0 | 0 | 0 | 1 | 0 |
|  | **Death** | 11 (5,5%) | 4 | 1 | 3 | 1 | 2 |
|  | **Lost to follow-up** | 1 (0,5%) | 0 | 0 | 1 | 0 | 0 |
|  | **Disease progression** | 22 (11,1%) | 3 | 6 | 5 | 6 | 2 |
|  | **Toxicity** | 31 (15,6%) | 8 | 1 | 5 | 11 | 6 |
|  | **Other** | 2 (1,0%) | 0 | 0 | 0 | 0 | 2 |

## Table S5. Adverse events classified according to CTC-AE 4.0

| N = 199 | Total | Grade  1 | Grade  2 | Grade  3 | Grade  4 | Grade unknown |
| --- | --- | --- | --- | --- | --- | --- |
| 1. Blood and lymphatic system disorders | **105 (52.8%)** | **59** | **27** | **17** | **2** | **0** |
| 2. Cardiac disorders | 2 (1.0%) | 0 | 1 | 0 | 1 | 0 |
| 3. Congenital, familial and genetic disorders | - | - | - | - | - | - |
| 4. Ear and labyrinth disorders | 3  (1.5%) | 3 | 0 | 0 | 0 | 0 |
| 5. Endocrine disorders | - | - | - | - | - | - |
| 6. Eye disorders | 2 (1.0%) | 2 | 0 | 0 | 0 | 0 |
| 7. Gastrointestinal disorders | **121 (60.8%)** | **55** | **54** | **12** | **0** | **0** |
| 8. General disorders and administration site conditions | **113 (56.8%)** | **47** | **51** | **13** | **1** | **1** |
| 9. Hepatobiliary disorders | - | - | - | - | - | - |
| 10. Immune system disorders | 6 (3.0%) | 0 | 3 | 2 | 1 | 0 |
| 11. Infections and infestations | 11 (5.5%) | 2 | 7 | 1 | 1 | 0 |
| 12. Injury, poisoning and procedural complications | 1 (0.5%) | 1 | 0 | 0 | 0 | 0 |
| 13. Investigations | **106 (53.3%)** | **38** | **31** | **25** | **12** | **0** |
| 14. Metabolism and nutrition disorders | **42 (21.1%)** | **27** | **11** | **3** | **0** | **1** |
| 15. Musculoskeletal and connective tissue disorders | **35 (17.6%)** | **16** | **17** | **1** | **0** | **1** |
| 16. Neoplasms benign, malignant and unspecified (incl. cysts and polyps) | - | - | - | - | - | - |
| 17. Nervous system disorders | **91 (45.7%)** | **40** | **35** | **15** | **1** | **0** |
| 18. Pregnancy, puerperium and perinatal conditions | - | - | - | - | - | - |
| 19. Psychiatric disorders | 1 (0.5%) | 0 | 0 | 0 | 1 | 0 |
| 20. Renal and urinary disorders | 5 (2.5%) | 4 | 0 | 1 | 0 | 0 |
| 21. Reproductive system and breast disorders | - | - | - | - | - | - |
| 22. Respiratory, thoracic and mediastinal disorders | **66 (33.2%)** | **61** | **4** | **0** | **0** | **1** |
| 23. Skin and subcutaneous tissue disorders | **88 (44.2%)** | **21** | **65** | **2** | **0** | **0** |
| 24. Social circumstances | - | - | - | - | - | - |
| 25. Medical and surgical procedures | - | - | - | - | - | - |
| 26. Vascular disorders | **27 (13.6%)** | **14** | **11** | **2** | **0** | **0** |

Bold letters highlight those with frequency >10%

## Table S6. Association between VEGF, VEGFR-2, CD31, CD34, CD133, CD146 levels and objective response to treatment

| **Treatment response** | ***VEGF-A*** | | | ***VEGFR-2*** | | | ***CD31*** | | | ***CD34*** | | | ***CD133*** | | | ***CD146*** | | |
| --- | --- | --- | --- | --- | --- | --- | --- | --- | --- | --- | --- | --- | --- | --- | --- | --- | --- | --- |
|  | **Low**  **n (%)** | **High n (%)** | ***p*** | **Low**  **n (%)** | **High n (%)** | ***p*** | **Low**  **n (%)** | **High n (%)** | ***p*** | **Low**  **n (%)** | **High n (%)** | ***p*** | **Low**  **n (%)** | **High n (%)** | ***p*** | **Low**  **n (%)** | **High n (%)** | ***p*** |
| **Disease progression** | 3 (5.2) | 4 (7.6) | 0.869 | 11 (47.8) | 36 (40.9) | 0.789 | 4 (8.2) | 1 (2.1) | 0.492 | 3 (6.4) | 2 (4.3) | 1.000 | 2 (4.7) | 1 (2.3) | 1.00 | 2 (4.7) | 2 (4.6) | 0.631 |
| **Stable disease** | 24 (41.4) | 23 (43.4) |  | 22 (40.0) | 25 (44.6) |  | 19 (38.8) | 19 (40.4) |  | 19 (40.4) | 19 (40.4) |  | 17 (39.5) | 18 (41.9) |  | 19 (44.2) | 15 (34.1) |  |
| **Partial response** | 31 (53.5) | 26 (49.1) |  | 30 (54.6) | 27 (48.2) |  | 26 (53.1) | 27 (57.5) |  | 25 (53.2) | 26 (55.3) |  | 24 (55.8) | 24 (55.8) |  | 22 (51.2) | 27 (61.4) |  |
